# Potential Therapeutic Benefit of Low Dose Naltrexone in Myalgic Encephalomyelitis/Chronic Fatigue Syndrome: Role of Transient Receptor Potential Melastatin 3 Ion Channels in Pathophysiology and Treatment

**DOI:** 10.3389/fimmu.2021.687806

**Published:** 2021-07-13

**Authors:** Helene Cabanas, Katsuhiko Muraki, Natalie Eaton-Fitch, Donald Ross Staines, Sonya Marshall-Gradisnik

**Affiliations:** ^1^ The National Centre for Neuroimmunology and Emerging Diseases, Menzies Health Institute Queensland, Griffith University, Gold Coast, QLD, Australia; ^2^ Consortium Health International for Myalgic Encephalomyelitis, National Centre for Neuroimmunology and Emerging Diseases, Griffith University, Gold Coast, QLD, Australia; ^3^ Laboratory of Cellular Pharmacology, School of Pharmacy, Aichi-Gakuin University, Nagoya, Japan

**Keywords:** low dose naltrexone, transient receptor potential melastatin 3, calcium, opioid receptor, myalgic encephalomyelitis/chronic fatigue syndrome, natural killer cells, whole-cell patch clamp electrophysiology

## Abstract

Myalgic Encephalomyelitis/Chronic Fatigue Syndrome (ME/CFS) is a debilitating multi-systemic chronic condition of unknown aetiology classified as an immune dysfunction syndrome and neurological disorder. The discovery of the widely expressed Transient Receptor Potential Melastatin 3 (TRPM3) as a nociceptor channel substantially targeted by certain opioid receptors, and its implication in calcium (Ca^2+^)-dependent Natural Killer (NK) cell immune functions has raised the possibility that TRPM3 may be pharmacologically targeted to treat characteristic symptoms of ME/CFS. Naltrexone hydrochloride (NTX) acts as an antagonist to the mu (μ)-opioid receptor thus negating its inhibitory function on TRPM3. Based on the benefits reported by patients on their symptoms, low dose NTX (LDN, 3.0–5.0 mg/day) treatment seems to offer some potential benefit suggesting that its effect may be targeted towards the pathomechanism of ME/CFS. As there is no literature confirming the efficacy of LDN for ME/CFS patients *in vitro*, this study investigates the potential therapeutic effect of LDN in ME/CFS patients. TRPM3 ion channel activity was measured after modulation with Pregnenolone sulfate (PregS) and ononetin in NK cells on 9 ME/CFS patients taking LDN and 9 age- and sex-matched healthy controls using whole-cell patch-clamp technique. We report that ME/CFS patients taking LDN have restored TRPM3-like ionic currents in NK cells. Small ionic currents with a typical TRPM3-like outward rectification were measured after application of PregS, a TRPM3-agonist, in NK cells from patients taking LDN. Additionally, PregS-evoked ionic currents through TRPM3 were significantly modulated by ononetin, a TRPM3-antagonist, in NK cells from ME/CFS patients taking LDN. These data support the hypothesis that LDN may have potential as a treatment for ME/CFS by characterising the underlying regulatory mechanisms of LDN treatment involving TRPM3 and opioid receptors in NK cells. Finally, this study may serve for the repurpose of marketed drugs, as well as support the approval of prospective randomized clinical studies on the role and dose of NTX in treating ME/CFS patients.

## Introduction

Myalgic Encephalomyelitis/Chronic Fatigue Syndrome (ME/CFS) is a severe multisystemic condition characterized by persistent debilitating fatigue accompanied by a range of disabling symptoms including post-exertional malaise, sleep dysfunction, widespread pain, as well as neuro-cognitive, autonomic, cardiovascular, neuroendocrine and immune manifestations (1, 2). There is varying severity of ME/CFS (mild through very severe) (3), where significant and consistent immune dysfunction and abnormalities in Natural killer (NK) cell functions have been reported based on severity (4). Without a clinically established diagnostic test, diagnosis is complex and imprecise. Diagnosis currently relies on different symptom-based case definitions, known as the Canadian Consensus Criteria (CCC, 2003) and the International Case Criteria (2011), which address the clinical characteristics of ME/CFS (2, 5, 6). The aetiology and specific pathophysiology of the condition, which affects 17 to 24 million people worldwide (7), remain unknown.

ME/CFS potentially develops because of impairment in Transient Receptor Potential (TRP) non-selective cation channels caused by either genetic or acquired factors (8–13). Previous studies suggested the importance of TRP Melastatin 3 (TRPM3), a widely expressed calcium (Ca^2+^)-permeable nonselective cation channel, in the pathophysiology of ME/CFS (8–10). Recently, independent electrophysiological investigations demonstrated a loss-of-function of TRPM3 ion channel after stimulation with two potent TRPM3-agonists, pregnenolone sulfate (PregS) and nifedipine, and inhibition with a selective and potent blocker, the deoxybezoin ononetin, in NK cells from three different cohorts of ME/CFS patients (11–13).

TRPM3 has been previously identified as a chemo- and thermosensitive nociceptor channel in the somatosensory system and is implicated in the detection of noxious heat and inflammatory heat hyperalgesia as well as in pain transmission (14). Recent literature suggests that signalling pathways associated with activation of certain G-protein coupled receptors (GPCRs), called opioid receptors, can inhibit TRPM3 ion channel activity by direct binding of the G_βγ_ subunit of the trimeric G-proteins to the ion channel (15–18). Physiologically, opioid receptors are activated by endogenous opioid peptides to modulate pain mechanisms and inflammatory pathways (19). The opioid drugs, or opiates produce both analgesia and undesirable side effects by attaching to the same GPCRs (19). Evidence indicates that opioid receptors are not only expressed by the central nervous system (CNS) but are widely distributed on tissues and organ systems outside the CNS, such as the immune cells (20). Activation of leukocyte opioid receptors leads to the secretion of endogenous opioid peptides, which subsequently activate neuronal opioid receptors and alleviate pain (21). Opioids were also shown to have immunosuppressive effects on the innate and adaptative immune system (22). ME/CFS pathology may be considered as an ion channel disorder or channelopathy (8–13) as well as an immune dysfunction syndrome and neurological disorder (23, 24). Thus, it is vital to investigate the regulatory mechanisms underlying the TRPM3-opioid receptor interaction in NK cells from ME/CFS patients to better understand the pathogenesis.

The discovery of TRPM3 as a nociceptor channel substantially targeted by certain opioid receptors, as well as its implication in Ca^2+^-dependent NK cell immune functions suggests that TRPM3 ion channel may be pharmacologically targeted to treat ME/CFS. Naltrexone hydrochloride (NTX) is a long-lasting opioid antagonist specifically targeting the mu-opioid receptor (μOR) and, to a lesser extent, the delta-opioid receptors (δOR) (25, 26). The binding of NTX on μORs negates the inhibitory function of these opioid receptors on TRPM3 (15, 17, 18, 26). We have previously reported that *in vitro* treatment of NK cells from ME/CFS patients with NTX for 24hrs, restores the TRPM3 ion channel activity (13). Restoration of TRPM3 ion channel activity in turn re-established the appropriate Ca^2+^ signalling and *per se* NK cells’ activation and effector functions (13). NTX is commonly used in the treatment of opioid and alcohol dependence in a dose of 50-100 mg/day (25). At high dosage, NTX is thought to block the euphoric feelings of beta-endorphins which are involved in natural reward circuits and therefore can sustain addiction (27). A low dose (3.0–5.0 mg/day, low-dose NTX, LDN) appears to have an opposite effect, enhancing the endorphin effect to reduce pain and boost pleasure (28). This is believed to have relevance in illnesses such as ME/CFS, where insufficient secretion of endogenous opioid peptides affects immune response or pain control (29, 30), or the release of pro-inflammatory cytokines (25, 31). A recent study reported that LDN is safe and effective to alleviate ME/CFS symptoms by retrospective examination of the clinical patient records of 218 ME/CFS patients administered LDN (27). Even if new evidence indicates that LDN can improve daily function and quality of life in ME/CFS patients, further *in vitro* investigation is needed to understand the underlying regulatory mechanisms involving TRPM3 and opioid receptors in NK cells to help understand the pathomechanism of ME/CFS and identify therapeutic targets.

This present study investigated the potential therapeutic effect of LDN in ME/CFS patients by restoring TRPM3 ion channel function in NK cells using whole cell patch-clamp techniques. Our results indicate that ME/CFS patients taking LDN have restored TRPM3-like ionic currents in NK cells. Restoration of TRPM3 function is required to promote Ca^2+^ signal remodelling in NK cells and *per se* NK cell activation and effector functions. This is the first study to report the efficacy of LDN for ME/CFS patients *in vitro*, therefore confirming potential for use as a treatment for the disease.

## Materials and Methods

### Participant Recruitment

Nine ME/CFS patients and nine age- and sex-matched healthy controls (HC) were recruited using the National Centre for Neuroimmunology and Emerging Diseases (NCNED) patient database between September 2019 and February 2021. Participants were aged between 18 and 60 years. All ME/CFS patients had previously received a confirmed medical diagnosis and were screened using a comprehensive online questionnaire corresponding with the Centers for Disease Control and Prevention (CDC), CCC and ICC case definitions. All nine ME/CFS patients were defined by the CCC. All nine ME/CFS patients were recruited by NCNED as they had previously started LDN treatment (3.0–5.0 mg/day) for at least 4 weeks as part of their management plan after prescription by their general practitioner. HC reported no incidence of fatigue and were in good health without evidence of illness. Participants were excluded from this study if they were pregnant or breastfeeding, or reported a previous history of smoking, alcohol abuse or chronic illness (for example, autoimmune diseases, cardiovascular disease, diabetes, metabolic syndrome, thyroid disease, malignancies, insomnia, chronic fatigue and primary psychological disorders) or were obese (BMI ≥ 30). No participants reported use of opioids or any other pain killers in the preceding 3 months as well as pharmacological agents that directly or indirectly influence TRPM3 or Ca^2+^ signalling. Participants were provided with the option to cease any conflicting medications for a minimum of 14 days prior to blood donation with the approval of their physician. All participants completed an online questionnaire to provide sociodemographic background, medical history, medications, and symptom history for ME/CFS patients (**Table 1**). The 36-item short form health survey (SF-36) and World Health Organization (WHO) Disability Assessment Schedule (DAS) were used to determine level of disability and quality of life (**Table 2**) (32, 33). Finally, ME/CFS patients were asked to self-report severity of symptoms prior and after taking LDN (**Table 3**). This investigation was approved by the Griffith University Human Research Ethics Committee (GU2019/1005) and Gold Coast University Hospital Human Research Ethics Committee (HREC/2019/QGC/56469).

**Table 1:** Participant demographics.

		HC	ME/CFS taking LDN	P-value
Age (years)		47.33 ± 4.64	48.33 ± 4.972	0.8849
Gender n(%)	Female	8 (88.9%)	8 (88.9%)	0.999
	Male	1 (11.1%)	1 (11.1%)	0.999
BMI (kg/m^2^)		24.61 ± 0.858	24.63 ± 1.227	0.8812
Work Status	Full Time	5 (55.6%)	1 (11.1%)	0.2433
	Part Time	3 (33.3%)	1 (11.1%)	
	Casual	1 (11.1%)	1 (11.1%)	
	Unemployed	0 (0%)	0 (0%)	
	Illness/Disability	0 (0%)	6 (66.7%)	
Education	Primary Education	0 (0%)	0 (0%)	**0.0045**
	High School	1 (11.1%)	2 (22.2%)	
	Undergraduate	1 (11.1%)	3 (33.3%)	
	Postgraduate/Doctoral	3 (33.3%)	2 (22.2%)	
	Other	4 (44.4%)	2 (22.2%)	

Participant age (years), gender, BMI (kg/m^2^). Work status and education was reported in Table 1. Data are presented as mean ± SEM. BMI, body mass index; HC, healthy controls; ME/CFS, myalgic encephalomyelitis/chronic fatigue syndrome; n, sample number.

Bold values are results that are statistically significant, set at p < 0.05.

**Table 2:** Participant quality of life, disability scores and serology.

	HC	ME/CFS	P-value
**SF-36** (%)			
Physical Functioning	93.33 ± 5.528	30.78 ± 7.297	**0.0002**
Physical Role	99.31 ± 0.6944	15.97 ± 8.597	**<0.0001**
Pain	91.94 ± 3.859	41.94 ± 7.117	**<0.0001**
General Health	83.33 ± 4.39	34.25 ± 6.544	**<0.0001**
Social Functioning	100 ± 0	19.44 ± 8.866	**<0.0001**
Emotional Role	100 ± 0	71.3 ± 12.51	**0.0090**
Emotional Wellbeing	79.94 ± 2.922	42.28 ± 3.577	**<0.0001**
**WHO DAS** (%)			
Communication & Understanding	2.78 ± 1.840	40.74 ± 4.436	**<0.0001**
Mobility	1.67 ± 1.66	51.11 ± 8.571	**<0.0001**
Self-Care	0 ± 0	25.0 ± 8.398	**0.0090**
Interpersonal Relationships	0 ± 0	34.72 ± 7.591	**0.0004**
Life Activities	0 ± 0	63.89 ± 10.77	**<0.0001**
Participation in Society	0.69 ± 0.69	55.17 ± 9.372	**<0.0001**
**Full Blood Count**			
White Cell Count (x10^9^/L)	5.79 ± 0.42	5.711 ± 0.6647	0.9223
Lymphocytes (x10^9^/L)	1.95 ± 0.23	2.039 ± 0.2975	0.8085
Neutrophils (x10^9^/L)	3.14 ± 0.23	3.05 ± 0.346	0.5457
Monocytes (x10^9^/L)	3.14 ± 0.25	0.453 ± 0.06	0.7819
Eosinophils (x10^9^/L)	0.19 ± 0.02	0.134 ± 0.03	**0.0333**
Basophils (x10^9^/L)	0.05 ± 0.004	0.04 ± 0.01	0.3532
Platelets (x10^9^/L)	304.1 ± 19.64	275.3 ± 15.47	0.2667
Red Cell Count (x10^12^/L)	4.38 ± 0.15	4.52 ± 0.177	0.5606
Haematocrit	0.41 ± 0.01	0.412 ± 0.01	0.7391
Haemoglobin (g/L)	132.6 ± 3.81	136.8 ± 4.71	0.4958

SF-36 and WHODAS scores were analysed using participant questionnaire responses. Results from routine full blood were analysed in ME/CFS patients taking LDN and HC. Data are presented as mean ± SEM. Abbreviations: HC, healthy controls; ME/CFS, myalgic encephalomyelitis/chronic fatigue syndrome; SF-36, 36-item short form survey; WHO, world health organization; DAS, disability assessment schedule.

**Table 3:** ME/CFS symptom characteristics.

Age of diagnosis (Years [Mean ± SD])				26.78 ± 10.44
Disease duration (Years [Mean ± SD])				22 ± 14.04
Infectious onset, n(%)				6 (66.7%)
Other onset, n(%)				3 (33.3%)
Duration since taking LDN (Month [Mean ± SD])				21.11 ± 24.66
Dosage of LDN (mg/day [Mean ± SD])				4.06 ± 0.68
		Prior n(%)	After n(%)	P-value
Impaired thought, concentration and cognitive overload	None	0 (0%)	0 (0%)	**0.004**
	Mild	0 (0%)	4 (44.4%)	
	Moderate	2 (22.2%)	4 (44.4%)	
	Severe	6 (66.7)	1 (11.1%)	
	Extreme	1 (11.%)	0 (0%)	
Memory consolidation issues	None	0 (0%)	0 (0%)	0.077
	Mild	1 (11.%)	5 (55.6%)	
	Moderate	5 (55.6%)	3 (33.3%)	
	Severe	2 (22.2%)	1 (11.1%)	
	Extreme	1 (11.%)	0 (0%)	
Sleep Disturbances	None	0 (0%)	(0%)	0.077
	Mild	0 (0%)	1 (11.1%)	
	Moderate	4 (44.4%)	7 (77.8%)	
	Severe	5 (55.6%)	1 (11.1%)	
	Extreme	0 (0%)	0 (0%)	
Muscle pain	None	1 (11.%)	2 (22.2%)	0.605
	Mild	3 (33.3%)	2 (22.2%)	
	Moderate	1 (11.1%)	2 (22.2%)	
	Severe	3 (33.3%)	3 (33.3%)	
	Extreme	1 (11.1%)	0 (0%)	
Joint pain	None	2 (22.2%)	2 (22.2%)	0.730
	Mild	3 (33.3%)	3 (33.3%)	
	Moderate	1 (11.1%)	3 (33.3%)	
	Severe	3 (33.3%)	1 (11.1%)	
	Extreme	0 (0%)	0 (0%)	
Sore throat	None	0 (0%)	1 (11.1%)	0.077
	Mild	2 (22.2%)	5 (55.6%)	
	Moderate	4 (44.4%)	2 (22.2%)	
	Severe	3 (33.3%)	1 (11.1%0	
	Extreme	0 (0%)	0 (0%)	
Tender lymph nods	None	1 (11.1%)	3 (33.3%)	0.136
	Mild	4 (44.4%)	5 (55.6%)	
	Moderate	3 (33.3%)	1 (11.1%)	
	Severe	1 (11.1%)	0 (0%)	
	Extreme	0 (0%)	0 (0%)	
Other immune disturbances	None	0 (0%)	1 (11.1%)	**0.024**
	Mild	0 (0%)	2 (22.2%)	
	Moderate	5 (55.6%)	6 (66.7%)	
	Severe	4 (44.4%)	0 (0%)	
	Extreme	0 (0%)	0 (0%)	
Gastrointestinal Disturbances	None	0 (0%)	0 (0%)	0.297
	Mild	3 (33.3%)	3 (33.3%)	
	Moderate	1 (11.1%)	5 (55.6%)	
	Severe	4 (44.4%)	1 (11.1%)	
	Extreme	1 (11.1%)	0 (0%)	
Urinary Disturbances	None	0 (0%)	3 (33.3%)	0.136
	Mild	5 (55.6%)	4 (44.4%)	
	Moderate	3 (33.3%)	2 (22.2%)	
	Severe	1 (11.1%)	0 (0%)	
	Extreme	0 (0%)	0 (0%)	
POTS	None	3 (33.3%)	3 (33.3%)	0.489
	Mild	0 (0%)	2 (22.2%)	
	Moderate	3 (33.3%)	3 (33.3%)	
	Severe	2 (22.2%)	0 (0%)	
	Extreme	1 (11.1%)	1 (11.1%)	
Thermostatic Instability	None	0 (0%)	0 (0%)	0.077
	Mild	1 (11.1%)	4 (44.4%)	
	Moderate	1 (11.1%)	3 (33.3%)	
	Severe	6 (66.7%)	1 (11.1%)	
	Extreme	1 (11.1%)	1 (11.1%)	

ME/CFS patients self-reported the severity of their symptoms prior and after taking LDN. Data are presented as mean ± SD or frequency n(%). HC, healthy controls; ME/CFS, myalgic encephalomyelitis/ chronic fatigue syndrome; n, sample number; POTS, Postural orthostatic tachycardia syndrome.

Bold values are results that are statistically significant, set at p < 0.05.

### Peripheral Blood Mononuclear Cell Isolation and Natural Killer Cell Isolation

A total of 85 ml of whole blood was collected in ethylendiaminetetraacetic acid (EDTA) tubes *via* venepuncture by a qualified phlebotomist from each participant between 8:30am and 10:30am at collection locations including Griffith University, Royal Brisbane and Women’s Hospital, Robina Hospital, Sunshine Coast University Hospital, Calvary John James Hospital, Royal Melbourne Hospital, Australia. Routine full blood analysis was performed within four hours of collection for red blood cell count, white blood cell count and granulocyte cell count for each participant at Gold Cost University Hospital or Royal Melbourne Hospital, Australia.

Samples were provided to the laboratory de-identified using a unique code by an independent blood collector. Peripheral blood mononuclear cells (PBMCs) were isolated from 80 ml of whole blood by centrifugation over a density gradient medium (Ficoll-Paque Premium; GE Healthcare, Uppsala, Sweden) as previously described (34, 35). PBMCs were stained with trypan blue (Invitrogen, Carlsband, CA, USA) to determine cell count and cell viability. PBMCs were adjusted to a final concentration of 5x10^7^ cells/ml for NK cell isolation.

NK cells were isolated by immunomagnetic selection using an EasySep Negative Human NK Cell Isolation Kit (Stem Cell Technologies, Vancouver, BC, Canada). NK Cell purification was determined using flow cytometry. NK cells were incubated for 20 minutes at room temperature in the presence of CD56 FITC (0.25µg/5µl) and CD3 PE Cy7 (0.25µg/20µl) monoclonal antibodies (BD Bioscience, San Jose, CA, USA) as previously described (10). 7-amino-actinomycin (7-AAD) (2.5µl/test) (BD Bioscience, San Jose, CA, USA) was used to determine cell viability. Cells were washed and resuspended in 200 μl of stain buffer (BD Bioscience, New Jersey, USA) and acquired at 10,000 events using the LSRFortessa X-20 (BD Biosciences, San Diego, CA, USA). Using forward and side scatter, the lymphocyte population was gated while acquiring the sample. The NK cell population was then identified as CD3^−^CD56^+^ cells.

### Whole Cell Electrophysiology Recording

Electrophysiological recordings were performed with borosilicate glass capillary electrodes with an outside diameter of 1.5 mm and inside diameter of 0.86 mm (Harvard Apparatus, Holliston, MA, USA). Pipette resistance when filled with pipette solution was 8-12 MΩ. The pipettes were mounted on a CV203BU head-stage (Molecular Devices, Sunnyvale, CA, USA) connected to a 3-way coarse manipulator and a micro-manipulator (Narishige, Tokyo, Japan). Electrical signals were amplified and recorded using an Axopatch 200B amplifier and PClamp 10.7 software (Molecular Devices, Sunnyvale, CA, USA). Data were filtered at 5 kHz and sampled digitally at 10 kHz *via* a Digidata 1440A analogue to digital converter (Molecular Devices, Sunnyvale, CA, USA). The voltage-ramp protocol was a step from a holding potential of +10 mV to −90 mV, followed by a 0.1 s ramp to +110 mV, before returning to +10 mV (repeated every 10 s). The liquid junction potential between the pipette and bath solutions (−10 mV) was corrected. A leak current component was not subtracted from the recorded currents. Electrode was filled with the intracellular pipette solution containing 30 mM CsCl, 2 mM MgCl_2_, 110 mM L-Aspartic acid, 1 mM EGTA, 10 mM HEPES, 4 mM ATP, 0.1 mM GTP, adjusted pH to 7.2 with CsOH and osmolality of 290 mOsm/L with D-mannitol. The pipette solution was filtered using a 0.22 μm membrane filter (Sigma-Aldrich, St. Louise, MO, USA), divided into aliquots and stored at −20 °C. Bath solution contained: 130 mM NaCl, 10 mM CsCl, 1 mM MgCl_2_, 1.5 mM CaCl_2_2H_2_O, 10 mM HEPES, adjusted pH to 7.4 with NaOH and osmolality 300 mOsm/L with D-glucose. All reagents were purchased from Sigma-Aldrich, except for ATP and GTP that were purchased from Sapphire Bioscience. TRPM3 ionic currents on NK cells were stimulated by adding 100 μM PregS (Tocris Bioscience, Bristol, UK) to the bath solution, whereas PregS-induced TRPM3 currents were blocked by adding 10 μM ononetin (Tocris Bioscience, Bristol, UK). For each participant, 10 different recordings were made. Hence, N refers to number of participants in each group (ME/CFS patients or HC) and n, to number of readings or sample size. All measurements were performed at room temperature. The authors removed the possibility of chloride current involvement in TRPM3 assessment by using L-Aspartic acid in the intracellular pipette solution. Cells which have unstable currents were also excluded from the analysis.

### Statistical Analysis

Lymphocyte populations were identified by flow cytometry using forward and side scatter dot plots. The purity of CD3^-^CD56^+^ lymphocytes was determined. Cytometry data was exported from FacsDiva v8.1 and analysed using SPSS v24 (IBM Corp, Version 24, Armonk, NY, USA) and GraphPad Prism v7 (GraphPad Software Inc., Version 7, La Jolla, CA, USA). Electrophysiological data were analysed using pCLAMP 10.7 software (Molecular Devices, Sunnyvale, CA, USA). Origin 2018 (OriginLab Corporation, Northampton, MA, USA) and GraphPad Prism v7 (GraphPad Software Inc., Version 7, La Jolla, CA, USA) were used for statistical analysis and data presentation. Visual and computed methods were used to determine normality of independent data. Specifically, histogram plots and the Shapiro-Wilk normality test, skewness and kurtosis were used to determine the distribution of data and normality. Depending on normality, statistical comparison was performed using the independent nonparametric Mann-Whitney U test, or independent parametric t-test. Fishers exact test was used to determine NK cells sensitivity to ononetin. Significance was set at p<0.05 and the data are presented as mean ± SEM unless otherwise stated.

## Results

### Participant Characteristics and Blood Parameters

Eighteen age- and sex-matched participants were included for this investigation. Demographic data for patients are summarised in **Table 1**. There was no significant difference in age or gender between ME/CFS patients and HC. The SF-36 and WHODAS surveys were used to determine participant health-related-quality of life (**Table 2**) (36, 37). As expected, there was a significant difference in SF-36 and WHODAS scores between ME/CFS patients and HC. Moreover, full blood count parameters were measured for each healthy participant and ME/CFS patients (**Table 2**). While we reported a significant difference for eosinophils between healthy participants and ME/CFS patients, all results were within the specified reference ranges as provided by the Gold Coast University Hospital Pathology Unit, NATA accredited laboratory. No significant differences were reported in other parameters. Finally, ME/CFS patients reported improvement in impaired thought, concentration and cognitive overload as well as other immune disturbances symptoms (sore throat, enlarged or tender lymph nodes, and susceptibility to colds/influenza) after treatment with LDN (**Table 3**), as described before (27, 38).

### Natural Killer Cell Purity

**Figure 1:**
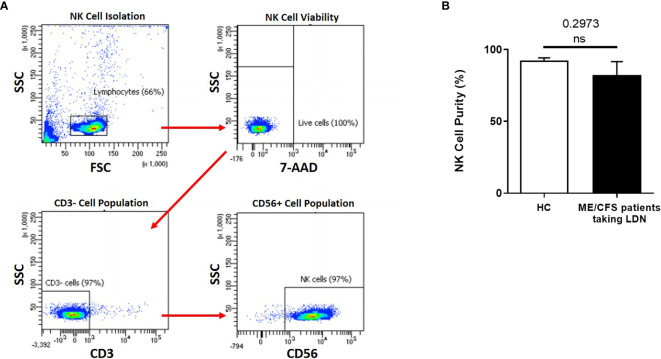
Natural Killer cell purity. **(A)** Gating strategy used to identify NK cells. Representative flow cytometry plots from the PBMCs of one of the study participants. The lymphocytes were live gated during acquisition using the side and forward scatter dot plot display and then single and dead cells were excluded. Furthermore, by using the negative and positive gating strategies, CD3^−^ as well as CD56^+^ lymphocyte populations were identified. **(B)** Bar graphs representing isolated NK cell purity for HC and ME/CFS patients taking LDN. Data presented as mean ± SEM. HC = 92.42% ± 1.849 and ME/CFS taking LDN= 82.20% ± 9.409. 7-AAD, 7-amino-actinomycin; FSC, forward scatter; HC, healthy controls; LDN, low dose naltrexone; ME/CFS, myalgic encephalomyelitis/chronic fatigue syndrome; NK cell, natural killer cell; SSC, side scatter. NS, not significant.

NK cell purity (CD3^-^/CD56^+^) was 92.42% ± 1.849 for HC and 82.20% ± 9.409 for ME/CFS patients taking LDN as determined by flow cytometry (**Figure 1A**). There was no significant difference in NK cell purity in ME/CFS patients taking LDN compared with HC (**Figure 1B**).

### TRPM3 Ion Channel Activity After Pregnenolone Stimulation in NK Cells From ME/CFS Patients Taking LDN Compared With NK Cells From HC

**Figure 2:**
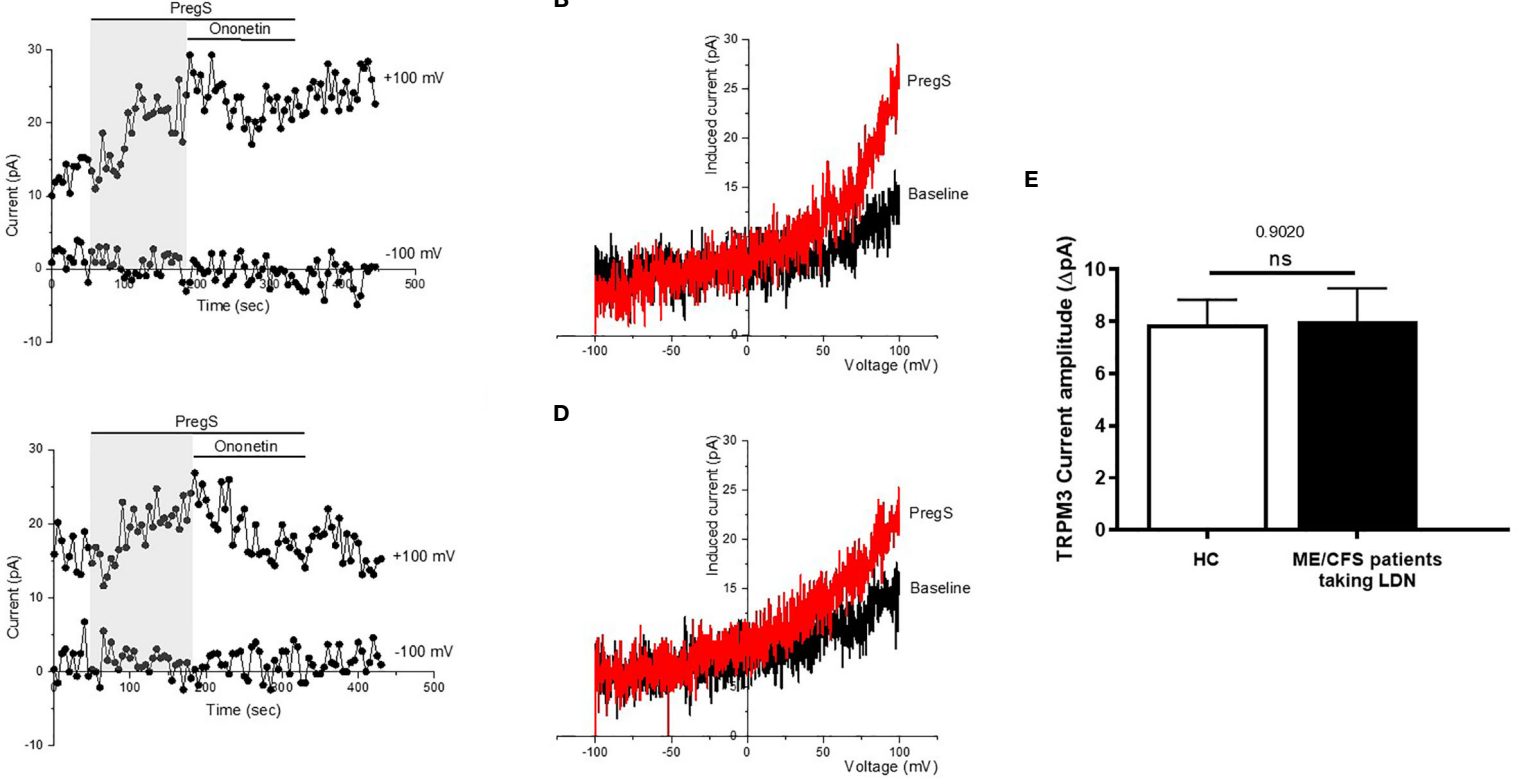
TRPM3 activity after PregS stimulation in NK cells from ME/CFS patients taking LDN compared to HC. Data were obtained under whole-cell patch clamp conditions. **(A)** A representative time-series of current amplitude at + 100 mV and − 100 mV showing the effect of stimulation with 100 mu;Mu; PregS (grey) on ionic currents in isolated NK cells from HC. **(B)** *I*–*V* before and after PregS stimulation in a cell corresponding with **(A)**. **(C)** A representative time-series of current amplitude at + 100 mV and − 100 mV showing the effect of stimulation with 100 mu;Mu; PregS (grey) on ionic currents in isolated NK cells from ME/CFS patients taking LDN. **(D)** *I*–*V* before and after PregS stimulation in a cell as shown in **(C)**. **(E)** Bar graphs representing TRPM3 current amplitude at + 100 mV after stimulation with 100 mu;Mu; PregS in isolated NK cells from ME/CFS patients taking LDN (*N = 9*; *n* = 49) compared with HC (N = 9; *n* = 56). Data are represented as mean ± SEM. HC, healthy controls; LDN, low dose naltrexone; ME/CFS, myalgic encephalomyelitis/chronic fatigue syndrome; NK, natural killer; PregS, Pregnenolone sulfate; TRPM3, Transient Receptor Potential Melastatin 3. NS, not significant.

TRPM3 ion channel activity was recorded in isolated human NK cells from ME/CFS patients taking LDN (3.0–5.0 mg/day) and compared to isolated human NK cells from age- and sex-matched HC, using whole-cell patch-clamp technique. Endogenous TRPM3 ion channel function was quickly and reversibly activated by application of 100 μM PregS, a neuronal steroid. Stimulation with PregS enabled measurement of a small outwardly rectifying current under voltage-clamp conditions and observation of a typical shape of the TRPM3 current–voltage relationship (*I*–*V*) (**Figure 2**). We therefore measured a small ionic current with a typical TRPM3-like outward rectification in NK cells isolated from HC after addition of PregS (**Figures 2A, B**). Interestingly, PregS application in NK cells from ME/CFS patients taking LDN mimicked the PregS-induced increase in NK cells from HC (**Figures 2C, D**). Thus, PregS stimulation induced an increase of the outwardly rectified current amplitudes in NK cells from ME/CFS patients taking LDN to the same level as NK cells from HC (**Figure 2E**). Moreover, the PregS-evoked currents present a typical *I*–*V* of TRPM3 (**Figure 2D**). The data suggest that ME/CFS patients taking LDN do not longer have an impaired TRPM3 channel activity as reported previously (11–13), and therefore LDN may have a potential therapeutic benefit for the pathology.

### Modulation of PregS- Evoked Currents With Ononetin in NK Cells From ME/CFS Patients Taking LDN Compared With NK Cells From HC

**Figure 3:**
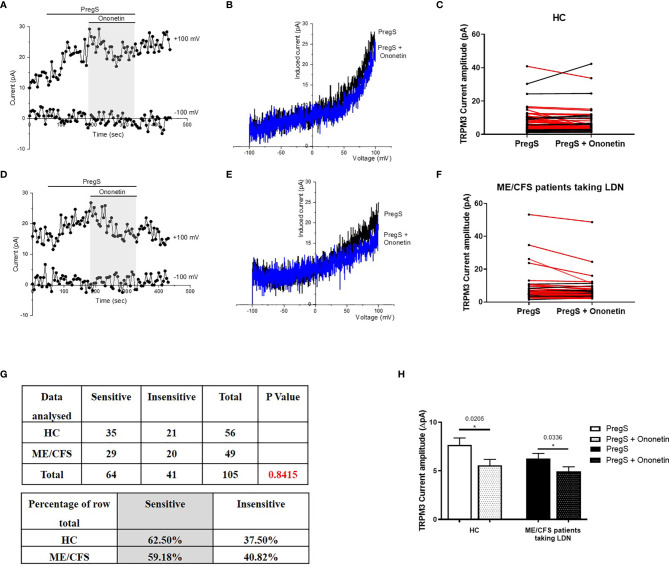
Modulation of PregS- evoked currents with Ononetin in NK cells from ME/CFS patients taking LDN compared to HC. Data were obtained under whole-cell patch clamp conditions. **(A)** A representative time-series of current amplitude at +100 mV and −100 mV showing the effect of 10 µM ononetin (grey) on ionic currents in the presence of 100 µM PregS in NK cells from HC. **(B)** *I***–***V* before and after application of ononetin in the presence of PregS in a cell as shown in **(A)**. **(C)** Scatter plots representing change of each current amplitude before and after application of ononetin in presence of PregS in all NK cells from HC. Each cell represented as red lines had reduction in currents by ononetin. **(D)** A representative time-series of current amplitude at +100 mV and −100 mV showing the effect of 10 µM ononetin (grey) on ionic currents in the presence of 100 µM PregS in NK cells from ME/CFS patients taking LDN. **(E)** *I***–***V* before and after application of ononetin in the presence of PregS in a cell as shown in **(D)**. **(F)** Scatter plots representing change of each current amplitude before and after application of ononetin in presence of PregS in all NK cells from ME/CFS patients taking LDN. Each cell represented as red lines had reduction in currents by ononetin. **(G)** Table summarizing data for sensitive and insensitive cells to the application of 10 µM ononetin in presence of PregS in ME/CFS patients taking LDN (*N = 9; n = 49*) compared to HC (*N = 9; n = 56*). Data are analysed with Fisher’s exact test. **(H).** Bar graphs representing TRPM3 current amplitude at + 100 mV after stimulation with 100 mu;Mu; PregS and application of 10 µM ononetin in presence of PregS in isolated NK cells from ME/CFS patients taking LDN (*N = 9*; *n* = 25) compared with HC (N = 9; *n* = 35). Only the records sensitive to ononetin have been included from **(G)**. The ROUT outlier test has been applied and any outliers identified have been removed. Data are represented as mean ± SEM. HC, healthy controls; LDN, low dose naltrexone; ME/CFS, myalgic encephalomyelitis/chronic fatigue syndrome; NK, natural killer; PregS, Pregnenolone sulfate.

PregS-induced Ca^2+^-influx and ionic currents through TRPM3 ion channels can be effectively inhibited by a natural compound, deoxybezoin ononetin (39). Therefore, to confirm that TRPM3 activity is involved in PregS-evoked ionic currents in NK cells, we next used 10 μM ononetin to modulate TRPM3 ion channel activity (**Figure 3**). As previously shown (11–13), the ionic currents evoked by application of PregS were effectively inhibited by simultaneous application of ononetin in isolated NK cells from HC (**Figures 3A–C**). Moreover, the *I*–*V* of ononetin sensitive currents was outwardly-rectified and typical for TRPM3 (**Figure 3B**). Interestingly, the ionic currents evoked by PregS application were also effectively inhibited by simultaneous application of 10 μM ononetin in isolated NK cells from ME/CFS patients taking LDN (**Figures 3D–G**). Indeed, TRPM3-dependent ionic current has been previously reported to be resistant to ononetin in isolated NK cells from ME/CFS patients (11–13). However, NK cells from ME/CFS patients taking LDN are sensitive to ononetin in the same range as NK cells from HC (**Figure 3G**). Moreover, the *I*–*V* of ononetin sensitive currents was outwardly-rectified and typical for TRPM3 (**Figure 3E**). Finally, **Figure 3H** reports TRPM3 current amplitudes after PregS stimulation and ononetin modulation within the HC and ME/CFS taking LDN groups and only the records sensitive to ononetin have been included. A significant decrease of TRPM3 current amplitude was reported after modulation with ononetin in both ME/CFS patients taking LDN (p=0.0336) and HC (p=0.0205) groups. This graph confirms TRPM3 ion channel functions similarly in ME/CFS patients taking LDN in comparison with HC as the same current amplitude ranges are reported (**Figure 3H**). Collectively, these results confirm that ME/CFS patients taking LDN do not have an impaired TRPM3 ion channel activity, which is reported to be characteristic of the disease (11–13). In conclusion, treatment with LDN allow to restore TRPM3 ion channel function in ME/CFS patients and has therefore a potential therapeutic effect.

## Discussion

In the present study, we used the whole-cell patch-clamp technique to measure the endogenous TRPM3 ion channel function in NK cells from ME/CFS patients taking LDN. We report for the first time that ME/CFS patients taking LDN have restored TRPM3-like ionic currents in NK cells. Indeed, application of PregS enabled the measurement of small ionic currents with a typical TRPM3-like outward rectification in NK cells from patients taking LDN. In addition, PregS-evoked ionic currents through TRPM3 were significantly modulated by ononetin in NK cells from ME/CFS patients taking LDN as similarly described in HC. These results reflect our previous findings reporting a restoration of TRPM3 ion channel function in NK cells from ME/CFS patients after *in vitro* treatment with NTX (13). These results are also consistent with our previous investigations suggesting ME/CFS may be considered as a channelopathy induced by TRPM3 loss-of-function and characterised by a decrease of the PregS-induced ionic currents as well as a resistance to ononetin in NK cells from ME/CFS patients (8–13). The TRPM3 loss-of-function may be responsible for impaired Ca^2+^ signalling and Ca^2+^-mediated cell functions, including NK cell immune functions (11–13).

Our present novel study is the first *in vitro* study confirming the efficacy and therapeutic benefit of LDN for ME/CFS patients by characterising the underlying regulatory mechanisms of LDN treatment involving TRPM3 and the opioid receptor interaction in NK cells. LDN has been previously reported to be beneficial for treatment of a variety of diseases, such as multiple sclerosis, Crohn’s disease, fibromyalgia, cancer, inflammatory bowel disease and ME/CFS (40–44). More specifically, in the preliminary study conducted by Polo et al. on the safety and effectiveness of LDN treatment, 73.9% of ME/CFS patients reported a positive treatment response (27). Most patients experienced improved vigilance/alertness and improved physical and cognitive performance. Some patients also reported less pain and fever, demonstrating that LDN may show some potential to alleviate a wide spectrum of ME/CFS symptoms (27). Similarly, Bolton and al. described three case reports compiled by ME/CFS patients taking LDN and reported the range of responses, from life changing to a reduction in some symptoms only (38). Based on the benefits reported by patients on their symptoms, LDN treatment may be used as potential treatment and may target towards the pathomechanism of ME/CFS.

The pathogenesis of ME/CFS is unknown but abnormal ion channels and more specifically impaired TRPM3 function have recently appeared as a characteristic feature of the illness (8–13). We have previously reported TRPM3 loss-of-function after modulation with potent TRPM3-agonists and antagonists in NK cells from three different cohorts of ME/CFS patients (11–13). TRPM3 has been described to be a central actor of Ca^2+^-dependent signalling pathways, in both excitable and non-excitable cells, where Ca^2+^ signals drive the different cellular processes (45). Papanikolaou et al, identified TRPM3 as an important component of the ‘Ca^2+^ toolkit’ that underpins store-operated Ca^2+^ entry and the sustainability of Ca^2+^ signalling in CNS white matter glia (46). Similarly, TRPM3 was shown to lead to an increase of Ca^2+^ influx in pancreatic beta cells resulting in subsequent insulin release (47). Each cell type presents a unique signalling phenotype capable of delivering tightly regulated spatiotemporal Ca^2+^ signals to modulate its particular function (48). Consequently, any subtle dysregulations of Ca^2+^ signals may highly impact cell functions and result in diseases (48, 49).

NK cells also require Ca^2+^ for efficient stimulation and functions including NK cell cytotoxicity (50–53). Compromised NK cell cytotoxic function is a well-documented and consistent feature of ME/CFS (54). Therefore, TRPM3 loss-of-function may contribute to ME/CFS symptomatology by affecting Ca^2+^ signalling and Ca^2+^-mediated cell functions, including NK cell immune functions.

A well-characterized function of TRPM3 in the literature is its role in in the detection of noxious heat (acute pain) as well as inflammatory hyperalgesia (acute and chronic pain) (14). Endogenous opioids are synthesized *in vivo* to modulate the physiological analgesic responses in inflammatory and neuropathic pain. Endogenous and exogenous opioids bind to the ORs, found in the periphery, at pre-synaptic and postsynaptic sites in the dorsal horn of the spinal cord, and in the brain stem, thalamus, and cortex, in what constitutes the ascending pain transmission system (55). Recent studies have reported that opioid receptors are also expressed by immune cells to perform immune cell-mediated opioid analgesia by producing endogenous opioid peptides, which involves Gαi/o–Gβγ–phospholipase C (PLC)–inositol 1,4,5-trisphosphate receptor (IP_3_R)– intracellular Ca^2+^ pathway. The released opioid peptides subsequently activate peripheral neuronal opioid receptors and alleviate pain (21). Opioids were also shown to directly modulate the functions of lymphocytes and other cells involved in host defence and immunity resulting in immunosuppressive effects (56, 57). More specifically, activation of OR by opioid alkaloids and peptides such as morphine and the endogenous peptides, including beta-endorphin and the dynorphin peptides, lead to a decreased number of macrophages, reduced leukocyte proliferation and migration to inflammatory sites, decreased phagocytotic activity of macrophages, decreased chemotaxis and superoxide production by neutrophils and macrophages, accelerated apoptosis of macrophages, weakened microbiological protection, and reduced adhesion of leukocytes and endothelial cells (58). Other negative effects have also been described in relation to mast cells, dendritic cells, and NK cells with a reduced NK cell cytotoxicity and cytokine production (22, 56, 59, 60). The immune cells, glia and neurons form therefore an integrated network that coordinates immune responses and modulates the excitability of pain pathways (61). Targeting the opioid receptors on the immune cells may result in changes in immune responses to promote the resolution of inflammation as well as modulation of pain in ME/CFS patients.

Interestingly, molecules that bind to and activate opioid receptors significantly reduce TRPM3-dependent pain. In response to acute activation by agonists, opioid receptors couple to the G_i/o_ proteins, which dissociate into G_αi/o_ and G_βγ_ subunits to interact with intracellular effectors (62). TRPM3 activity is strongly inhibited by the activation of µOR through the direct interaction with G_βγ_ as TRPM3-dependent Ca^2+^ entries are strongly and reversibly inhibited when the cell membrane is flushed with purified G_βγ_ (15–18). NTX is a long-lasting opioid antagonist specifically inhibiting μORs, thus negating the inhibitory function of this opioid receptor on TRPM3 (22, 26). In our previous study, we have reported that *in vitro* treatment with NTX restores TRPM3 ion channel activity in NK cells isolated from ME/CFS (13). Similarly, in the current study, NK cells isolated from patients taking LDN did not exhibit any impaired TRPM3 ion channel activity. LDN treatment in ME/CFS patients appears to restore TRPM3 ion channel function in NK cells, resulting in Ca^2+^ signals remodelling to contribute to the restoration of the integrity and stability of the NK cell-specific signalling systems (10–13). Interestingly, it has been reported that the antagonism effect of NTX on μOR also disrupts the negative feedback regulation on δOR, thereby increasing δOR function. Enhancement of δOR activity promotes NK cell cytotoxicity in response to beta-endorphins *in vitro* on rat splenocytes, suggesting the potential use of NTX in the treatment of immune deficiency (63).

The mechanism underlying LDN’s efficacy for fatigue, Crohn’s disease, fibromyalgia, and multiple sclerosis therefore involves intermittent blockade of opioid receptors followed by Ca^2+^ dependent-production and upregulation of endogenous opioids. Ludwig et al. reported that serum [Met^5^]-enkephalin levels were lower in humans with multiple sclerosis relative to non-multiple sclerosis patients, and LDN restored their levels. Similarly, in experimental autoimmune encephalomyelitis mice, [Met^5^]-enkephalin levels were depressed prior to the appearance of clinical disease, and were restored with LDN treatment (28). Therefore, it seems that LDN treatment results in the enhancement of the analgesic effects and may be relevant in ME/CFS, where insufficient secretion of opioid peptides affects immunes response or pain control (29, 30), or the release of pro-inflammatory cytokines (25, 31). Indeed, reduced beta-endorphins concentration has been reported in ME/CFS patients suffering from dysregulation of thermoregulatory responses and generalized severe or long-lasting pain (64).

In conclusion, this study for the first time assessed and identified the effect of LDN on TRPM3 ion channel function in NK cells in ME/CFS patients. This novel study reports the underlying restoration of TRPM3-opioid receptor channel interaction in NK cells from ME/CFS patients taking LDN. Moreover, the wide distribution of TRPM3 ion channels in the body suggests that their compromised function may contribute to signs and symptoms of ME/CFS and therefore treatment with LDN may have a therapeutic role. Finally, this study serves to support the planning and approval of prospective randomized clinical studies on the role and dose of NTX in treating ME/CFS patients.

## Data Availability Statement

The original contributions presented in the study are included in the article/supplementary material. Further inquiries can be directed to the corresponding author.

## Ethics Statement

This investigation was approved by the Griffith University Human Research Ethics Committee (GU2019/1005) and Gold Coast University Hospital Human Research Ethics Committee (HREC/2019/QGC/56469). The patients/participants provided their written informed consent to participate in this study.

## Author Contributions

HC, KM, SM-G, and DS designed the study and wrote the manuscript. HC performed experiments. HC and KM performed data analysis. NE-F assisted with analysis. All authors contributed to the article and approved the submitted version.

## Funding

This study was supported by the Stafford Fox Medical Research Foundation (489798), the National Health and Medical Research Council (1199502), the McCusker Charitable Foundation (49979), the Buxton Foundation (4676), the Henty Community (4879), the Henty Lions Club (4880), the Mason Foundation (47107), the Mr Douglas Stutt, the Blake Beckett Trust Foundation (4579), the Alison Hunter Memorial Foundation (4570) and the Change for ME Charity (4575).

## Conflict of Interest

The authors declare that the research was conducted in the absence of any commercial or financial relationships that could be construed as a potential conflict of interest.

## Abbreviations

7-AAD, 7-amino-actinomycin; δOR, Delta opioid receptor; μOR, Mu opioid receptor; BMI, Body Mass Index; Ca^2+^, Calcium; CCC, Canadian Consensus Criteria; CDC, Centers for Disease Control and Prevention; CNS, Central nervous system; EDTA, Ethylendiaminetetraacetic acid; FBS, Fetal bovine serum; GPCRs, G-protein coupled receptors; HC, Healthy controls; ICC, International Consensus Criteria; IP_3_R, Inositol 1,4,5-trisphosphate receptor; *I-V*, Current–voltage relationship; LDN, Low Dose Naltrexone; ME/CFS, Myalgic Encephalomyelitis/Chronic Fatigue Syndrome; NCNED, National Centre for Neuroimmunology and Emerging Diseases; NK, Natural killer; NTX, Naltrexone hydrochloride; PBMCs, Peripheral blood mononuclear cells; PLC, Phospholipase C; POTS, Postural orthostatic tachycardia syndrome; PregS, Pregnenolone sulfate; RPMI, Roswell Park Memorial Institute medium; SF-36, 36-Item Short Form Survey; TRP, Transient receptor potential; TRPM3, Transient receptor potential melastatin 3; WHODAS, World Health Organization Disability Assessment Schedule.
